# Chip-Scale Ultra-Low Field Atomic Magnetometer Based on Coherent Population Trapping

**DOI:** 10.3390/s21041517

**Published:** 2021-02-22

**Authors:** Hyun-Gue Hong, Sang Eon Park, Sang-Bum Lee, Myoung-Sun Heo, Jongcheol Park, Tae Hyun Kim, Hee Yeon Kim, Taeg Yong Kwon

**Affiliations:** 1 Time and Frequency Group, Korea Research Institute of Standards and Science, Daejeon 34113, Korea; parkse@kriss.re.kr (S.E.P.); lsbum@kriss.re.kr (S.-B.L.); hms1005@kriss.re.kr (M.-S.H.); tykwon@kriss.re.kr (T.Y.K.); 2 Department of Convergence Sensor, National NanoFab Center, Daejeon 34141, Korea; jcpark@nnfc.re.kr (J.P.); thk@nnfc.re.kr (T.H.K.); hyeounkim@nnfc.re.kr (H.Y.K.)

**Keywords:** optical magnetometry, coherent population trapping, quantum sensor, chip-scale atomic device

## Abstract

We report a chip-scale atomic magnetometer based on coherent population trapping, which can operate near zero magnetic field. By exploiting the asymmetric population among magnetic sublevels in the hyperfine ground state of cesium, we observe that the resonance signal acquires sensitivity to magnetic field in spite of degeneracy. A dispersive signal for magnetic field discrimination is obtained near-zero-field as well as for finite fields (tens of micro-tesla) in a chip-scale device of 0.94 cm^3^ volume. This shows that it can be readily used in low magnetic field environments, which have been inaccessible so far in miniaturized atomic magnetometers based on coherent population trapping. The measured noise floor of 300 pT/Hz^1/2^ at the zero-field condition is comparable to that of the conventional finite-field measurement obtained under the same conditions. This work suggests a way to implement integrated atomic magnetometers with a wide operating range.

## 1. Introduction

Optically pumped atomic magnetometers are the most sensitive room-temperature magnetic sensors, which rely on precision measurements of atomic spin states under magnetic fields [1]. The sensitivity surpassing fT/Hz^1/2^ level has been reported in a spin-exchange-free regime (SERF) [2], and the absence of cryogenic cooling makes it favorable for a wide range of applications, such as tracking of magnetic objects [3,4], space exploration [5], and medical signal detection [6,7,8]. In particular, miniaturization based on microfabricated vapor cells [9,10,11] paves the way towards mass-producible low-power versions of atomic magnetometers, namely chip-scale atomic magnetometers (CSAMs). Recently CSAMs have been integrated with platforms of real-world applications, such as military systems [12] and wearable medical-imaging instruments [13,14]. Moreover, by exploiting the fact that atom-based measurements provide absolute values via fundamental relations of quantum mechanics, the chip-scale atomic reference platform with in-situ calibration capability have been envisioned [15].

Coherent population trapping (CPT) resonance [16] between two magnetically sensitive sublevels in hyperfine ground states of Alkali atoms is a well-known mechanism used to implement atomic magnetometers [17]. Although the sensitivity is not as high as that of magnetometers, based on direct detection of Larmor precession, the CPT scheme has its advantages in robustness and simplicity. For example, since it generally requires a less number of coils, one may expect reduced difficulties related to alignment and crosstalk [18,19]. In the perspective of heterogeneous integration of multiple devices [15,20], it can share major resources, such as a gigahertz-modulated laser with a chip-scale atomic clock (CSAC). Indeed, the first CSAM was realized on a physics package having the same architecture as the first CSAC [9]. However, to separate the magnetically sensitive transition from the clock transition by many linewidths, the measured field had an offset of ~70 μT, and this has been the case for almost all other CPT-based magnetometers [21,22].

Magnetic sensing near-zero-field, on the other hand, is desirable in some cases where the target field to be measured can be perturbed by the field generated by sensors. Coupling between sensors in an array configuration is also minimized for smaller field. In addition, biomagnetic measurement for medical diagnosis often requires a highly shielded environment. There has been a previous attempt to utilize degenerate CPT spectra near zero magnetic field for magnetometers [23], but not in a chip-scale package.

Here we report the CPT-based magnetic sensing near zero magnetic field using a millimeter-scale microfabricated vapor cell integrated in a full spectroscopy package of 0.94 cm^3^. Even in the regime where the linear Zeeman shift is much smaller than the CPT linewidth, we observed a Lorentzian microwave spectrum of 12.1 kHz linewidth, which is the result of combined resonances associated with all magnetic sublevels. The field inhomogeneity in this densely packed assembly was kept low enough so that the superimposed CPT resonance could work as a field discrimination signal under the nulled internal magnetic field. The magnetic sensitivity of the resonance, which arises from the asymmetric population among magnetic sublevels, is compared with the conventional finite-field (10.9 μT and 24.9 μT in this work) measurement based on a single Zeeman-shifted transition. The noise level of the field spectral density amounts to 300 pT/Hz^1/2^, which is comparable to the finite-field measurement in the same package and operation conditions. Consequently, by just tuning the microwave frequency to the linear Zeeman shift associated with the target field, we obtain a dynamic range of the CSAM extended down to zero-field.

This paper is organized as following. We first briefly describe the theoretical expectation in Section 2, and the sensor design and associated experimental settings in Section 3. We then present CPT spectra and the sensitivity analysis in Section 4, and summarize the result in Section 5.

## 2. Theory

**Figure 1:**
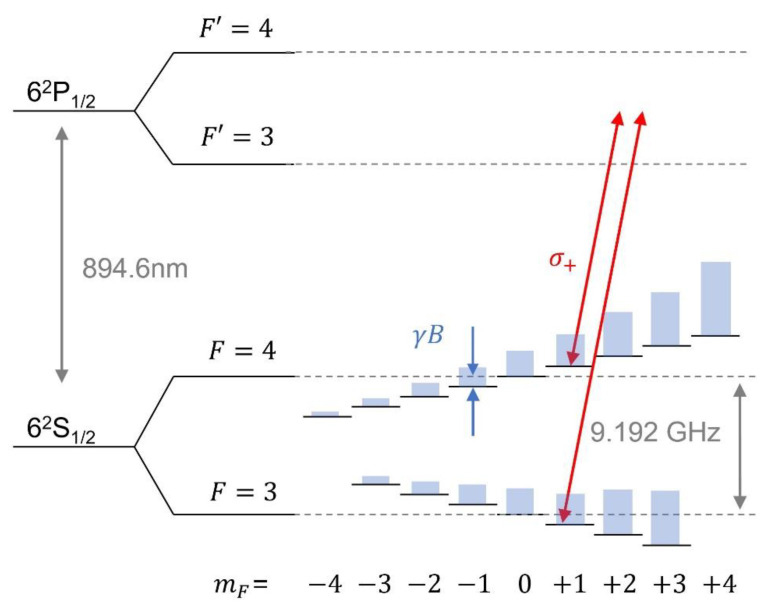
Energy level diagram of cesium D1 line with the (conceptual) steady state population of each magnetic sublevel expressed as height of blue bars. A circularly polarized light (red) is illustrated to create dark state resonance between $m_{F\;}$ = 1 states as an example. Likewise, each magnetic sublevels can be coupled by an appropriately tuned bichromatic field. Note that the modulation frequency should be half of 9.192 GHz if the bichromatic fields come from two first-order sidebands of a frequency-modulated laser as in our case.

Although it is possible to describe the system using a full quantum mechanical model [24], it suffices here to deal with simplified analytic formulae in order to obtain an intuitive picture. The CPT spectrum in its simplest configuration is obtained by measuring the laser transmission through atomic vapor, while the frequency difference of a bichromatic field is scanned across the transition frequency of hyperfine ground state [25]. We assume that the total spectrum is comprised of constituent Lorentzian resonances each of which corresponds to the transition between magnetic sublevels. The quantization axis is parallel to the direction of laser propagation, and we only consider the two-photon transitions by which the magnetic quantum number $m_{F}$ in the ground states does not change [26]. Each pair of sublevels is coupled with a bichromatic laser field via excited states in which individual magnetic sublevel is not resolved due to collisional and Doppler broadening. One may here refer to the energy level diagram of cesium shown in Figure 1, although our discussion is not limited to specific atomic species. By further assuming that the field inhomogeneity is negligible for simplicity and giving a common linewidth (full width at half maximum, FWHM) of Γ to all resonances, the spectrum reads

(1) $$S\left(\Delta \omega \right)\;=\;\overset{M}{\underset{k\;=\;-M}{\sum }}\frac{a_{k}}{{\left(\Delta \omega -2k\gamma B\right)}^{2}+\Gamma^{2}/4}$$

where Δω is the microwave frequency detuning from $m_{F\;}$ = 0 resonance, $a_{k}$’s represent the relative amplitude of each resonance, γ is the gyromagnetic ratio, B is the applied magnetic field, and M is the largest $m_{F}$ possible. In the limit of small magnetic field where γB is much smaller than Γ, the overlapped spectrum can be approximately given as

(2) $$\begin{array}{l} S\left(\Delta \omega \right)\approx \frac{1}{\Delta \omega^{2}+\Gamma^{2}/4}\left(\overset{M}{\underset{k\;=\;-M}{\sum }}a_{k}\right)+\frac{4\gamma B\Delta \omega }{{\left(\Delta \omega^{2}+\Gamma^{2}/4\right)}^{2}}\left(\overset{M}{\underset{k\;=\;1}{\sum }}ka_{k}+\overset{-1}{\underset{k\;=\;-M}{\sum }}ka_{k}\right)\\ \equiv \frac{P}{\Delta \omega^{2}+\Gamma^{2}/4}+\Delta \omega \frac{Q}{{\left(\Delta \omega^{2}+\Gamma^{2}/4\right)}^{2}} \end{array}$$

where, in the last line, P and Q are defined as the sum and difference of $a_{k}$’s. In principle, there can be complicated dynamics among sublevels as all possible transitions occur at the same time, but it turns out that this incoherent sum of spectra well explains experimental observations presented in Section 4. During the zero-field magnetometer operation, the microwave frequency is kept near the peak of the spectrum, thereby Δω≪Γ can also be applied. Thus, the approximated spectrum near Δω≈0 is expressed as a peak shifted by Q/P as the following equation

(3) $$S\left(\Delta \omega \right)\approx -\frac{P}{16\Gamma^{4}}{\left(\Delta \omega -\frac{Q}{P}\right)}^{2}+\frac{Q}{32P\Gamma^{4}}+\frac{4P}{\Gamma^{2}}$$

where the shifted peak position, Q/P, can be explicitly written as $4\gamma B\times \left(\sum_{k\;=\;1}^{M}ka_{k}+\sum_{k\;=\;-M}^{-1}ka_{k}\right)/\sum_{k\;=\;-M}^{M}a_{k}$, which is not necessarily zero depending on the relative amplitude of $a_{k}$’s. In fact, the simplest CPT configuration considered here includes only a single beam of circularly polarized light ($\sigma_{+}$ or $\sigma_{-}$), which leads to asymmetric population among the magnetic sublevels owing to optical pumping effect [24,27]. In a typical vapor-cell experiment, the factor $\left(\sum_{k\;=\;1}^{M}ka_{k}+\sum_{k\;=\;-M}^{-1}ka_{k}\right)/\sum_{k\;=\;-M}^{M}a_{k}$ is order of unity, which means that degradation of magnetic sensitivity is not serious unless detrimental effects, such as field inhomogeneity and misalignment distort the superimposed CPT signal.

## 3. Experimental Details

**Figure 2:**
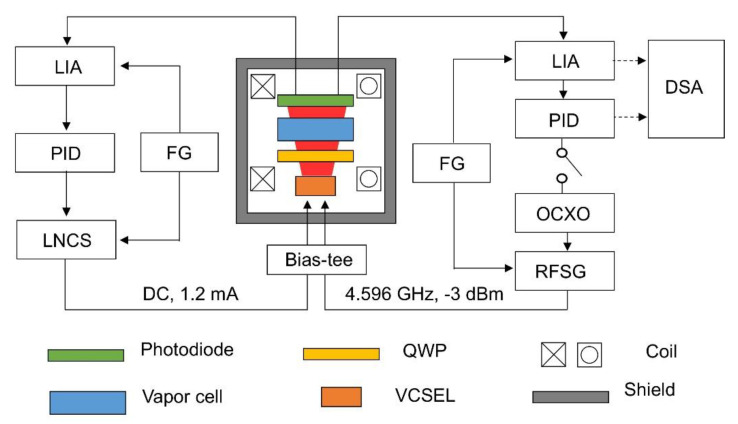
Schematic of the magnetometer package and measurement setup. Inside the shield is the spectroscopy unit and the instruments to control it are depicted outside the shield.

**Figure 3:**
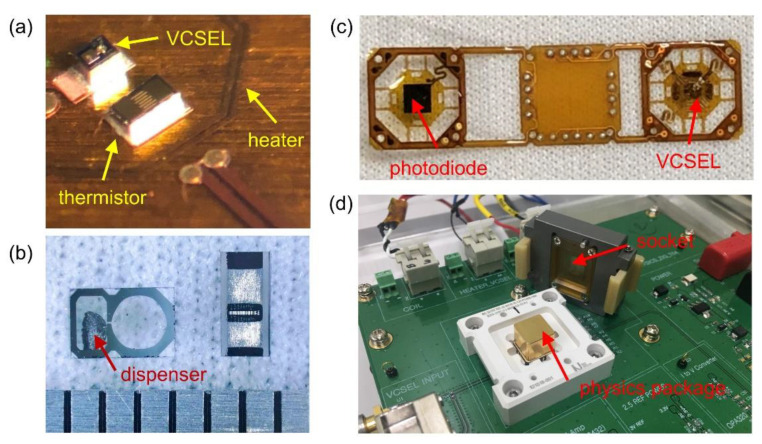
(**a**) Optical image of the substrate on which the VCSEL and the elements for temperature control are mounted. (**b**) The micro-fabricated vapor cell with a separate room for a cesium dispenser, which is activated by an infrared laser after bonding. (**c**) Flexible printed circuit board frame holding polyimide suspensions. (**d**) The printed circuit board with a socket used for interfacing the signal from the physics package.

Our chip-scale magnetometer package is basically identical to the one we used for a compact CPT clock [28]. The core spectroscopy unit is comprised of a vertical cavity surface emission laser (VCSEL), a quarter wave plate (QWP), a micro-fabricated cesium vapor cell, and a photodiode all aligned vertically (Figure 2). The current fed to the VCSEL chip is directly modulated so that its spectrum contains multiple sidebands, which are separated by 4.596 GHz from each other. The two first-order sidebands are tuned to induce dark-state resonance between the ground states via the excited state of D1 optical transition (Figure 1). The laser beam, which is linearly polarized at the VCSEL output, becomes circularly polarized upon passing through the QWP. It is a single crystal quartz substrate with a coating on one side for power attenuation. Then the laser transmission through the vapor cell is recorded in a photodiode. The vapor cell (Figure 3b), which measures 2 × 3 × 1.5 mm^3^, contains cesium metal, and is filled with neon buffer gas (270 Torr) to avoid wall collision. Due to the collisional broadening by the buffer gas, the Doppler-broadened linear absorption to F’ = 3 and F’ = 4 excited states are merged as a single peak. A pair of coils is built within the package in the axial direction, which is used to create the field to be measured or null out the residual field to set up a zero-field environment. It is patterned on a flexible printed circuit board in Helmholtz configuration. The amplitude of the magnetic field as a function of current injected into the coil is calibrated by using the linear Zeeman coefficient [29], which converts frequency shift to field amplitude, and it turns out to be 9.3 μT per 1 mA. Both the VCSEL and vapor cell are maintained near 80 °C, and they are thermally isolated by polyimide bridge structure (Figure 3c). To minimize internal magnetic noise induced by current paths near the atom, micro-fabricated elements for temperature sensing and heating are designed to cancel the magnetic field generated from the adjacent current path, and are located only on the substrate holding the VCSEL (Figure 3a). The vapor cell, which is 2 mm away from VCSEL, is thermally conducted via a silicon spacer. The power consumption for the heater, which accounts for most of the device operation power, is about 170 mW. The package is hermetically sealed in a 0.94 cm^3^ volume where the electric signal paths are interfaced via a ceramic chip carrier to a circuit board. The board distributes signals to off-the-shelf instruments for measurement and control (Figure 3d). We employ a compact magnetic shield, which allows us easy characterization of the package [28]. Although imperfect, it could be used, after nulling, for providing a zero-field environment due to small field-inhomogeneity as revealed in the linewidths of CPT spectra for high $m_{F}$’s. The nulling condition is determined by measuring Zeeman shift for various bias coil currents. In particular, the center of the quadratic Zeeman shift corresponds to the offset field [28], which amounts to 3.02 μT in this case. The zero-field environment is provided by nulling the residual field by the bias coil. We proceed our analysis hereafter by considering the net field at the vapor cell with the compensated offset taken into account.

The frequency of VCSEL is stabilized to the Doppler broadened absorption profile using a lock-in amplifier (LIA, Femto LIA-MVD-200-H) and a servo controller (Proportional–Integral–Derivative or PID, custom-made) as shown in Figure 2. The direct current (DC) input of VCSEL is provided from a low-noise current source (LNCS, custom-made) while the microwave input comes from a radio frequency signal generator (RFSG, Stanford Research SG386) piloted by an oven-controlled crystal oscillator (OCXO, NEL DFRM). The CPT signal is obtained by using another LIA (Stanford Research SR865) and a frequency modulation–demodulation method.

For closed-loop operation, the servo controller (Stanford Research SIM960) stabilizing the microwave frequency is enabled and the feedback signal is analyzed by a dynamic signal analyzer (DSA, Stanford Research SR785). For open-loop operation, the servo controller is disabled and the signal from LIA is directly recorded by the DSA.

## 4. Results

**Figure 4:**
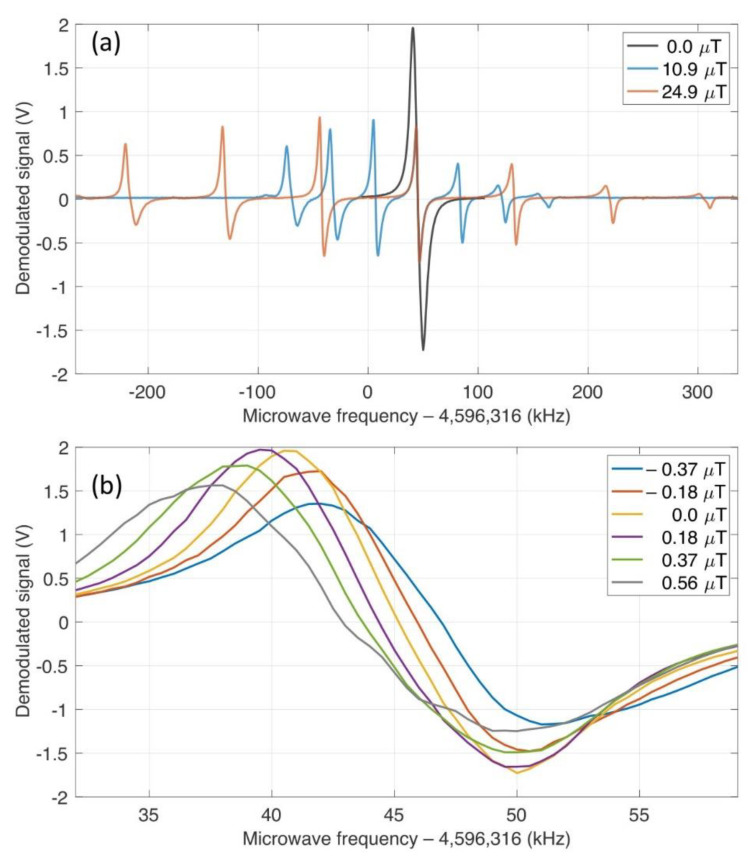
(**a**) Coherent population trapping (CPT) spectra as a function of microwave frequency for finite-field measurement (blue for 10.9 μT and red for 24.9 μT) and zero-field measurement (black) showing all possible resonances between magnetic sublevels given the magnetic field. (**b**) CPT spectra for a series of small magnetic fields varying near 0 μT.

In this section, we investigate the resulting spectrum and corresponding field measurement. The typical CPT microwave spectra for sufficiently large fields of 10.9 μT and 24.9 μT are shown in Figure 4a. There are well-resolved seven resonances, which originate from magnetic sublevels ranging from $m_{F}\;=\;$−3 to +3 with the change of$\;m_{F}$ being zero (Figure 1). The central one corresponds to the so-called clock transition, which is insensitive to magnetic field in the first order. Its resonance frequency has an offset from the exact half of ideal clock transition frequency (4,596,316 kHz) by ~45 kHz due to combined effects of light shift, pressure shift, second-order Zeeman shift etc. The linewidth (FWHM) of the clock transition is 6.8 kHz while other resonances are broadened by ~1.76 kHz$\;\times |m_{F}|$ due to field inhomogeneity.

**Figure 5:**
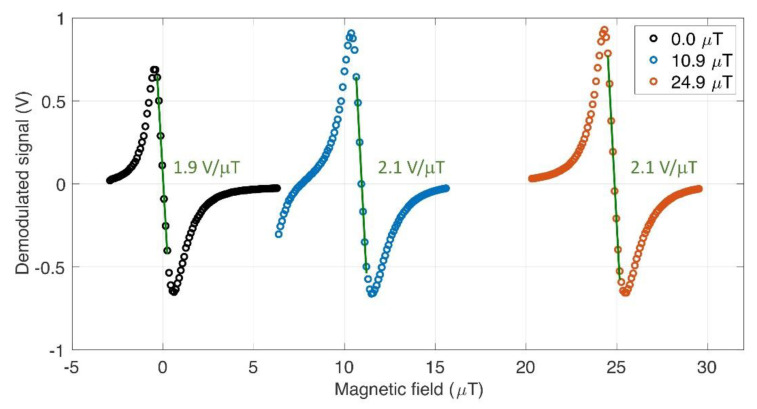
The lock-in amplifier (LIA) signal as a function of the axial magnetic field applied to the atom. The microwave frequency is tuned to measure each target field of 0.0 μT (black), 10.9 μT (blue), and 24.9 μT (red). Green curve: linear fitting near zero-crossing for each dispersive curve.

For conventional CPT-magnetometry, one of the magnetically sensitive resonances with significant amplitude is chosen as a discriminator for the magnetic field. Since the amplitude of the signal itself for $\;m_{F}\;$ = 2, 3 is smaller than those of $\;m_{F}\;\;$ = 0, 1 due to the broadening, we chose $\;m_{F}$ = 1 transition for our finite-field measurement. The absence of signal associated with transitions of different $m_{F}$’s indicates that the bias field is well-aligned to the laser propagation direction. As mentioned in Section 2, the asymmetry with respect to the clock transition is apparent due to the optical pumping by circularly polarized light. The field discrimination signal is obtained from the demodulated LIA output by sweeping the bias magnetic field with the microwave frequency fixed near each resonance associated with $m_{F}$ = 1. Both signals for 10.9 μT and 24.9 μT show the same level of discrimination, which is 2.1 V/μT as given by linear fitting (Figure 5).

Next, we consider the case near-zero-field. The CPT spectrum shown in Figure 4a is comprised of a single dispersion curves, which is the result of accumulation of resonance signals, thereby about two times larger in amplitude than that of $m_{F}\;$ = 1 resonance used for finite-field measurement. The linewidth of the merged resonance is 12.1 kHz, which is only two-times broadened compared to that of the magnetically insensitive clock transition.

The variation of resonance frequencies with respect to the magnetic field near 0 μT is shown in Figure 4b. The frequency sensitivity measured by the shift of zero-crossing is 3.7 kHz/μT, which is about the same as that of finite-field measurement for $m_{F}$ = 1 (3.5 kHz/μT) [29]. Based on the simple expression in Equation (3), we can estimate the frequency shift upon magnetic field variation by extracting $a_{k}$’s from data shown in Figure 4a. The estimation gives 6.5 kHz/μT, which is 1.85 times more sensitive than the measured value. Since we assumed the same linewidth of Γ for each resonance in the simple theory, we attribute this discrepancy to additional broadening for larger $m_{F}$’s, which brings about imperfect overlap of each resonance.

As the field deviate from the exactly nulled condition, the overlap of the degenerate resonance becomes imperfect, and consequently leads to reduction of the signal amplitude. The magnetic field discrimination signal obtained by scanning the bias field is also compared to those of finite field magnetometry in Figure 5. The slope of 1.9 V/μT is again similar to that of finite-field magnetometry under the same experimental conditions except the value of microwave frequency. The amplitude of the CPT signal itself is not as large as that in the frequency-scanned signal due to the aforementioned imperfection in the overlap of degenerate signals as the field deviates from zero. Based on the CPT analysis so far, we expect the similar level of noise sensitivity for the ultra-low field measurement to that of the conventional finite-field measurement.

**Figure 6:**
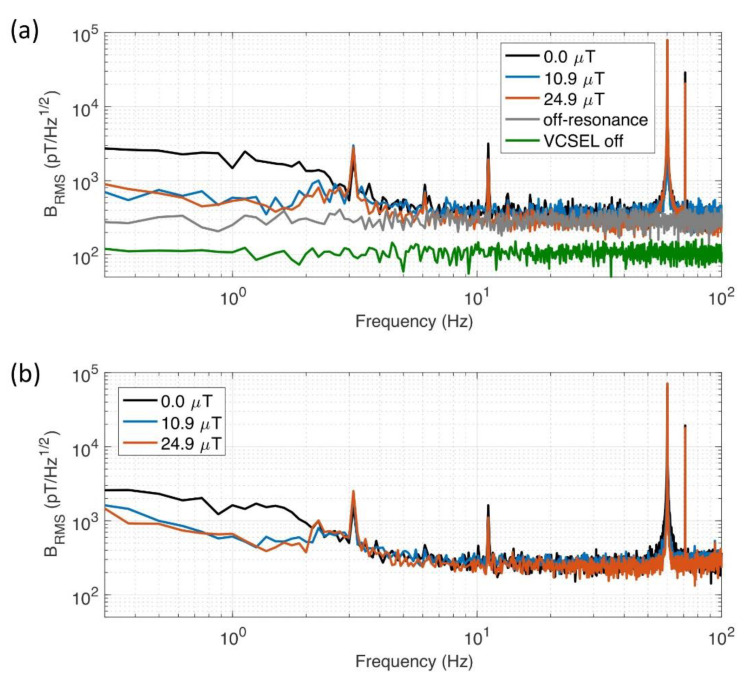
(**a**) Field noise spectral density obtained by Fourier analysis of the LIA output in the open loop mode. Black: 0.0 μT, blue: 10.9 μT, and red: 24.9 μT. For reference purpose, the results for completely off-resonant microwave frequency (gray) and in the absence of laser light (green) are overlaid. (**b**) Field noise spectral density obtained from the feedback signal of PID servo controller in the closed loop-mode. Same color code as (**a**) for target magnetic fields.

Now we present the magnetic field spectrum measured in both the open-loop mode and closed-loop mode to discuss the sensitivity of our CSAM, which shows an extended dynamic range. The spectrum in the open-loop mode is obtained by Fourier analysis of LIA signal with the servo being inactive. About 300 pT/Hz^1/2^ of noise floor above tens of Hertz is identified for all three target magnetic fields, including zero (Figure 6a). By detuning the microwave frequency far enough to avoid any CPT resonance (~1 MHz), we could verify this floor for all frequency ranges down to DC (gray curve in Figure 6a). The low frequency feature is the result of ambient magnetic field penetrated through the shield, and varies depending on the magnetic environment in the vicinity of the sensing module. The electronic noise limit is measured to be 100 pT/Hz^1/2^ by turning off the VCSEL current (green curve in Figure 6a). The result of closed-loop measurement (servo bandwidth ~1 kHz) obtained by stabilizing the microwave frequency to the peak of CPT resonance shows the consistent result as in Figure 6b. We attribute the large peaks at 11 Hz and 60 Hz to electric noise from instruments.

We believe that there is room for further improving the sensitivity of this magnetometer. First, it would be favorable to achieve higher density of atoms by increasing the cell temperature. In fact, the temperature of microfabricated vapor cell is around 100 °C for most CSAMs based on cesium [18,30]. Secondly, the CPT linewidth itself can be improved by reducing the laser beam divergence if we implement a micro-lens in the package. In our case, the diverging beam with a half angle of 20° passes through the cell. For a collimated beam, the intrinsic linewidth of CPT signal as low as 2 kHz has been obtained by using the same VCSEL and vapor cell in a separate tabletop experiment (not shown). Lastly, since the magnetic sensitivity comes from the asymmetric population, increasing the optical pumping efficiency is another way for improvement.

## 5. Conclusions

In conclusion, we demonstrated the operation of a CPT-based chip-scale magnetometer near zero magnetic field in a miniaturized package. In spite of densely assembled components in our chip-scale device of 0.94 cm^3^ in volume, the field inhomogeneity induced by internal parts was small enough to preserve the dispersive CPT spectra, even when they are all superimposed in the absence of magnetic field. We observed a similar level of magnetic sensitivity of resonance frequency to that of the individual CPT resonance of nonzero $m_{F}$ even in the ultra-low field environment. We also analyzed performances of the zero-field and finite-field magnetometers, both of which are comparable under the same condition. Further improvements are expected by optimizing the temperature and beam divergence. This work demonstrates successful operation of CPT-based CSAMs in a field range, which has been considered inaccessible. It also paves a way to further augment the simplicity of CSAMS, for example, by enabling operation without bias coils.

## Data Availability

The data presented in this study are available on request from the corresponding author.
